# Magnetic Micromanipulation for *In Vivo* Measurement of Stiffness Heterogeneity and Anisotropy in the Mouse Mandibular Arch

**DOI:** 10.34133/2020/7914074

**Published:** 2020-06-22

**Authors:** Min Zhu, Kaiwen Zhang, Hirotaka Tao, Sevan Hopyan, Yu Sun

**Affiliations:** ^1^Department of Mechanical and Industrial Engineering, University of Toronto, Canada M5S 3G8; ^2^Program in Developmental and Stem Cell Biology, Research Institute, The Hospital for Sick Children, Toronto, ON, Canada M5G 0A4; ^3^Department of Molecular Genetics, University of Toronto, Canada M5S 1A8; ^4^Division of Orthopaedic Surgery, The Hospital for Sick Children and University of Toronto, Canada M5G 1X8; ^5^Institute of Biomaterials and Biomedical Engineering, University of Toronto, Canada M5S 3G9; ^6^Department of Electrical and Computer Engineering, University of Toronto, Canada M5S 3G4

## Abstract

The mechanical properties of tissues are pivotal for morphogenesis and disease progression. Recent approaches have enabled measurements of the spatial distributions of viscoelastic properties among embryonic and pathological model systems and facilitated the generation of important hypotheses such as durotaxis and tissue-scale phase transition. There likely are many unexpected aspects of embryo biomechanics we have yet to discover which will change our views of mechanisms that govern development and disease. One area in the blind spot of even the most recent approaches to measuring tissue stiffness is the potentially anisotropic nature of that parameter. Here, we report a magnetic micromanipulation device that generates a uniform magnetic field gradient within a large workspace and permits measurement of the variation of tissue stiffness along three orthogonal axes. By applying the device to the organ-stage mouse embryo, we identify spatially heterogenous and directionally anisotropic stiffness within the mandibular arch. Those properties correspond to the domain of expression and the angular distribution of fibronectin and have potential implications for mechanisms that orient collective cell movements and shape tissues during development. Assessment of anisotropic properties extends the repertoire of current methods and will enable the generation and testing of hypotheses.

## 1. Introduction

The generation of tissue shape has long been recognized as a mechanical process [1, 2]. Increasingly, the elastic and viscous properties of tissues and forces generated by cells have been implicated in morphogenetic processes. The precision of these implications has been improving as we move from hypothetical assertions [3] to relative measurements [4], to absolute measurements [5–7] coupled with theory [7–9]. With regard to forces, the magnitude and directional bias, or polarity, of cytoskeletal contractions are relevant to how cells rearrange and bias the shapes of tissue sheets and bulk mesenchymal structures [5, 10–14]. The magnitudes and spatial distributions of tissue properties also influence the growth and form of tissues by mechanisms that are being elucidated [9, 11, 14]. Outstanding questions are whether and how tissue properties regulate anisotropic processes such as convergent extension. Convergent extension is a fundamental morphogenetic process that narrows and elongates many different tissues during multiple stages of development (e.g., gastrulation, neurulation, axis elongation, and organogenesis) [15]. It is unclear whether directionally biased or anisotropic viscoelastic properties promote or result from convergent extension, owing largely to the lack of tools to measure anisotropic properties *in vivo*.

Stiffness describes the ability of an object to resist deformation under external load. In biological tissues, the elastic modulus (a measure of tissue stiffness) varies by a few orders of magnitude, from a few hundreds of pascals (e.g., the brain) to a few gigapascals (e.g., cortical bone) [16]. In addition to providing structural support, tissue stiffness plays key roles in morphogenesis by regulating differentiation, proliferation, and migration [5, 7, 9, 14, 17–19]. For example, our recent analysis revealed a spatial stiffness gradient in the developing mouse limb bud that corresponds to the cell migration pattern (i.e., durotaxis), supporting the concept that heterogeneous tissue stiffness regulates cell behavior [7].

Besides heterogeneity of the magnitude of stiffness, tissues such as blood vessels, muscles, tendons, and bones exhibit anisotropy, i.e., they are stiffer along their load-bearing direction than other axes. *In vitro* studies showed that the anisotropy of substrate stiffness drives distinct cell behaviors such as directional growth, directional migration, and differentiation [20–22]. However, it remains unclear whether anisotropic stiffness exists in embryonic tissue and drives morphogenesis.

Several techniques have been applied to measure tissue stiffness. Atomic force microscopic (AFM) indentation is the most widely used method to measure surface or ectodermal tissue stiffness [5, 14, 18, 19]. Although AFM indentation has been employed to measure deeper mesodermal tissue stiffness, it requires either complex and controversial mechanical modelling and mathematical deconvolution [14] or surgical removal of the overlying ectoderm [19] which is suboptimal. In addition, as AFM indentation can only load the tissue surface perpendicularly, the sample is commonly assumed to be isotropic when extracting the stiffness values from experimental data via a mechanics model (e.g., Hertz model). However, the isotropic assumption is inappropriate since cells are known to deform anisotropically under AFM indentation [23], and the distribution of extracellular matrix (ECM) proteins in tissue is also anisotropic [24].

Optical and magnetic tweezers are untethered techniques that permit direct stiffness measurement inside tissue, and both are capable of applying forces in different directions. However, optical tweezers are limited to generating low forces (e.g., tens of piconewtons) due to potential tissue damage by laser power dissipation [25]. Therefore, optical tweezers have only been applied to measure properties *in vitro* [26–28] and in a limited capacity within epithelial tissue of *Drosophila* [29]. Magnetic tweezers, configured with either a single pole or multiple poles, have been used to measure cellular [30–32] and intracellular properties [33, 34]. Anisotropic, two-dimensional properties of cultured cells were revealed using a magnetic twisting device [32] and a single-pole magnetic tweezer [31]. Recently, magnetic tweezers were employed *in vivo* to measure tissue properties in *Drosophila* [35], *Zebrafish* [6], and preimplantation mouse embryos [36, 37]. Using AFM indentation, we had identified spatial and stage-dependent differences in the magnitude of stiffness within the mandibular arch [14] and limb bud [5] of the mouse embryo. More recently, we developed a multipole magnetic tweezer device that generates a uniform magnetic field gradient and applied it to identify a spatially more refined gradient of stiffness within the mesenchyme of the limb bud [7]. In both of these appendages, the isotropic versus anisotropic nature of stiffness remains unclear.

The mandibular arch of the mouse embryo is a useful model system in which to examine mechanical tissue properties because it is an accessible structure that acquires a distinct teardrop morphology, and we have characterized the epithelial and mesenchymal cell rearrangements which shape the tissue. Oriented, largely centripetal cell intercalations elongate and prevent radial expansion of the narrow proximal/middle region, while a lack of cell rearrangements are associated with bulbous expansion of the distal region [14]. Although cell rearrangements take place in a region of relatively low stiffness by AFM, any potential relationship between anisotropic tissue properties and the orientation of cell intercalations or of the curvature of the arch toward the midline is unclear.

In this work, we developed a new multipole magnetic tweezer device that generates a uniform magnetic field gradient within a fivefold larger workspace compared to the previous iteration [7]. Magnetic beads were deposited into the mandibular arch to measure three-dimensional tissue stiffness and determine both the spatial heterogeneity and the anisotropy of that property. In order to measure stiffness along three orthogonal axes, an E9.25 mouse embryo was rotated within the workspace of the magnetic tweezer device. The uniform magnetic field gradient ensured that the magnetic force applied to all the magnetic beads at different locations in the mandibular arch was identical and independent of position shifts of the beads during embryo rotation. Since the thickness of the mandibular arch is approximately 400 *μ*m, we integrated the magnetic device on a two-photon microscope stage for deeper near-infrared (NIR) imaging of the magnetic beads. Using this system, we confirmed that the distal region is stiffer than the middle region and identified stiffness anisotropy within the distal region as opposed to the isotropic stiffness within the middle region. The stiffness heterogeneity and anisotropy are aligned with the fibronectin expression domain and the orientation of the fibronectin fibers, respectively. These unexpected observations allow us to generate hypotheses about the influence of anisotropic tissue stiffness upon cell rearrangements and tissue curvature.

## 2. Results

### 2.1. Magnetic Device for *In Vivo* Tissue Stiffness Measurement

**Figure 1:**
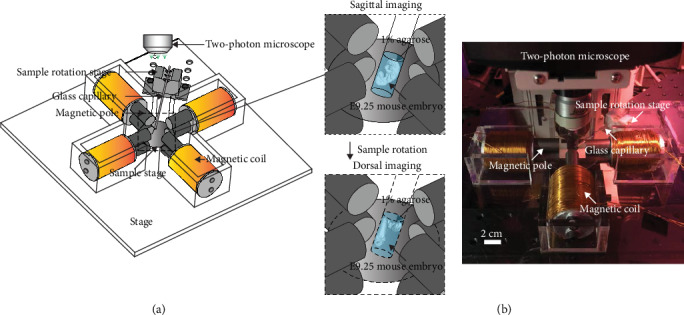
3D magnetic tweezer system. (a) Magnetic tweezer device, device stage, sample stage, and sample rotation stage with a glass capillary. The zoom-in view illustrates an E9.25 mouse embryo embedded in 1% agarose under examination in the sagittal view then rotated to be examined in the dorsal view. (b) Experimental set-up of the magnetic device mounted on a two-photon confocal microscope.

**Figure 2:**
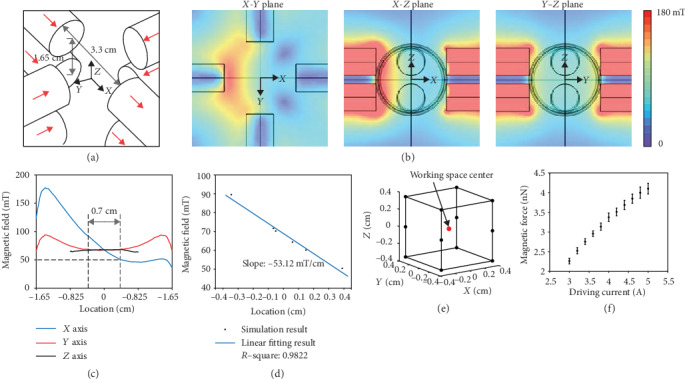
3D magnetic device characterization. (a) Configuration of magnetic poles. Red arrows indicate magnetic flux direction along each pole. (b) Simulated magnetic field under 3 A actuation plotted in *X*‐*Y*, *X*‐*Z*, and *Y*‐*Z* planes as shown in (a). (c) Simulated magnetic field along *X*, *Y*, and *Z* axes as shown in (a). The two vertical dash lines indicate a linear magnetic field in the *X* axis (i.e., uniform magnetic field gradient). (d) Linear fitting result of the region shown in (c). (e) Uniform magnetic field gradient workspace. (f) Experimentally characterized magnetic force-driving current data. Error bars represent s.d.

The magnetic tweezer device consists of four coils with eight magnetic poles (Figures 1(a)1(b) and 2(a)). The poles were vertically aligned in two layers, and the vertical layer-to-layer separation (1.65 cm) is half of the pole-to-pole separation (3.3 cm) in each layer. The magnetic field generated by the device was simulated using COMSOL Multiphysics. The simulated magnetic field under a 3 A driving current for each coil is shown in Figure 2(b). The simulated magnetic field is linear in the *X* axis and constant in the *Y* and *Z* axes within the workspace of a 0.7 cm cube (Figures 2(c)–2(e)).

For tissue stiffness measurement, magnetic beads were microinjected into the mandibular arch of E9.25 (20 som.) WT mouse embryos. The embryo was immersed in 1% agarose gel and held inside a glass capillary. A uniform magnetic field gradient in the *X*‐*Y* plane was generated to displace the magnetic beads in the mandibular arch for measuring tissue stiffness in four directions (±*X* and ±*Y*). No force was generated in the *Z* axis to ensure accurate measurement of bead displacements by two-photon microscopy. To measure tissue stiffness in the *Z* axis, the glass capillary with the mouse embryo inside was rotated by 90 degrees via a rotation stage to change the orientation of the tissue (Figure 1). At each orientation of the tissue, the penetration depth of the two-photon microscope (Leica DM6000) was sufficient to accurately measure displacements of the magnetic beads. The uniform magnetic field gradient ensures that the force applied by each magnetic bead is identical regardless of the beads' different positions within the mandibular arch or their position shifts caused by embryo rotation.

Magnetic force was calibrated by dispersing magnetic beads in silicone oil with known viscosity and applying current to the coils to actuate the beads (see Materials and Methods). Magnetic force was balanced by the fluidic drag force, according to Stokes equation, *F*_drag_ = 3*πdηυ*, where *d* is the diameter of the bead, *η* is the viscosity of the silicone oil, and *υ* is the velocity of the beads. To ensure that the magnetic force exerted on all beads is identical under the uniform magnetic field gradient, the magnetic beads must be saturated, requiring the magnetic field to be greater than 50 mT, which corresponds to at least a 3 A driving current in each coil of our magnetic tweezer device (see Materials and Methods). We chose to use relatively large magnetic beads (diameter: 32 *μ*m, Spherotech, SVMH-400-4) in this work because they are able to generate sufficiently large magnetic forces, effectively displace tissue, and reflect more global tissue properties. The deviation of the experimentally measured magnetic force (Figure 2(f)) from simulation results was less than 5% throughout the workspace. The magnetic beads used in this work were polystyrene containing ~12% iron oxide. The density of a magnetic bead was 1172 kg/m^3^. Since embryonic tissue density is similar to that of water, the net force (gravity-buoyancy) applied on the bead was ~29 pN which is orders of magnitude less than the nN level magnetic force and therefore was neglected from the measurement. The heat generated by the magnetic tweezer device was measured by a thermocouple probe. At a 5 A driving current for each coil and 10 s continuous actuation, the temperature increase at the workspace center was less than 1°C (Supplementary Fig. 1a), far below the threshold for potential thermal damage to tissue [38].

### 2.2. Mandibular Arch Stiffness Quantification

**Figure 3:**
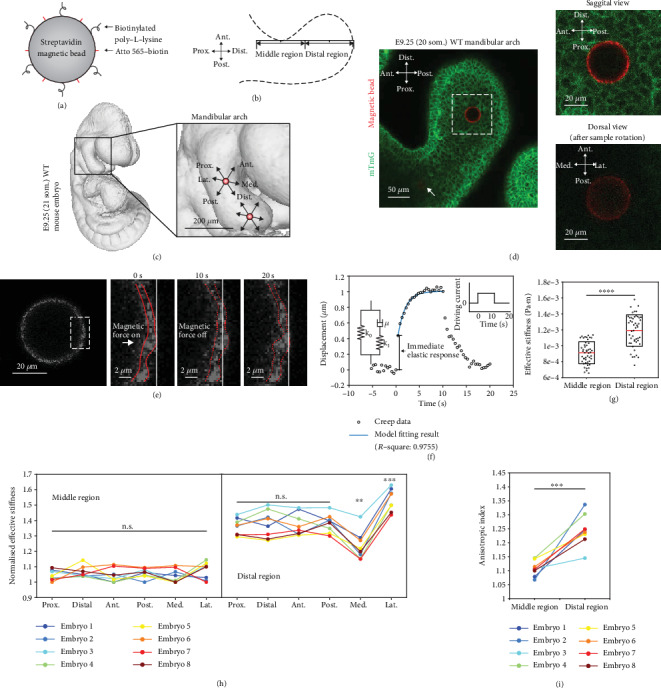
Mandibular arch stiffness quantification. (a) Schematic depicting magnetic bead surface functionalization. (b) Sketch describing the definition of the middle region and the distal region of the mandibular arch. (c) 3D rendering of optical projection tomography of an E9.25 WT (21 som.) mouse embryo. Zoom-in view illustrates the mandibular arch with two magnetic beads deposited in the center of the middle region and the distal region. (d) Two magnetic beads within mandibular mesenchyme. The magnetic bead in the middle region (arrow labelled) and the magnetic bead in the distal region were located at different depths in the mandibular arch because of the difference in thickness of the middle and distal regions. The zoom-in shows the magnetic bead in the sagittal view (before rotation) and the dorsal view (after rotation). (e) Higher magnification view of the magnetic bead before actuation. The zoom-in views show the displacement of the magnetic bead during one complete actuation cycle. The red dashed lines indicate the edges of the magnetic beads identified by the subpixel tracking algorithm. The white solid line indicates the original location of the magnetic bead. (f) Representative bead displacement of one actuation cycle (right inset) characterized by an immediate elastic response followed by a continuous creep which plateaus near the end of force application. The blue line shows the standard linear solid model (left inset) fitting result. (g) Effective stiffness of the middle and distal regions of E9.25 (20 som.) WT mouse mandibular arch (*n* = 8 embryos; two-tailed *t*-test, ^∗∗∗∗^*P* < 0.0001). (h) Normalized effective stiffness of the middle and distal regions measured in proximal, distal, anterior, posterior, medial, and lateral directions (paired two-tailed *t*-test, ^∗∗^*P* < 0.01, ^∗∗∗^*P* < 0.001). (i) Anisotropic index of measured stiffness in the middle and distal regions (paired two-tailed *t*-test, ^∗∗∗^*P* < 0.001).

For visualization under the two-photon microscope, all cell membranes were labelled green by crossing transgenic mTmG (membrane-localized tdTomato, membrane EGFP) embryos with ubiquitously expressed pCX-NLS-Cre [39]. Magnetic bead surfaces were fluorescently labelled by Atto 565 via streptavidin-biotin reaction. Biotinylated poly-l-lysine, a cell membrane adhesion molecule, was also coupled onto the surface of the magnetic beads to provide strong binding between a bead and the surrounding tissue (Figure 3(a)). The distal region of the mandibular arch was defined to span two-thirds of the total distance between the narrowest point and the distal end, and the middle region was taken to be of the same length as the distal region and centered at the narrowest point (Figure 3(b)). The middle region and the distal region each had a single 32 *μ*m magnetic bead deposited in their centers via microinjection (Figures 3(c) and 3(d)). Note that the magnetic bead in the middle region (see arrow in Figure 3(d)) and the magnetic bead in the distal region were located at different depths in the mandibular arch because of the difference in thickness of the middle and distal regions. Microinjection of magnetic beads did not result in detectable apoptosis as assessed by LysoTracker staining (Supplementary Fig. 2a), and in our previous study, injection had no effect on tissue morphology, cell death, or proliferation [7]. We therefore infer that mechanical tissue properties were not significantly altered by the process.

The magnetic beads were actuated for 10 s, and bead displacement was continuously measured until 10 s after magnetic actuation was turned off. Based on previously reported cell migration speeds in the mouse mandibular arch [14], migration-related cell displacement is less than 30 nm within 20 s and thus was neglected from stiffness quantification. As shown in Figure 3(d), tissue stiffness was first measured in the anterior, posterior, proximal, and distal directions. The mouse embryo was then rotated 90 degrees to the dorsal side, and tissue stiffness was then measured in the lateral and medial directions. The position of the magnetic bead upon actuation was measured by a subpixel tracking algorithm (Figure 3(e)) with a resolution of 0.2 pixels (pixel size: 0.37 *μ*m) as benchmarked by performing tracking of artificially generated circles on synthetic test images and as validated by tracking magnetic beads embedded in polyacrylamide (PA) gel (Supplementary Fig. 2b). The displacement of the magnetic beads upon actuation was due to the exerted magnetic force, which was confirmed by performing actuation on magnetic beads versus nonmagnetic beads embedded in PA gels (Supplementary Fig. 2c). When driving current was applied to the coils, a magnetic force was instantaneously exerted on the tissue, and the mandibular arch tissue exhibited an immediate elastic response followed by continuous creep (Figure 3(f)). After force removal, the tissue gradually recovered to its original shape. The stiffness value was extracted by fitting experimental data with a standard model commonly used for describing tissue behavior [6] (*R*‐square: 0.9755), as shown in Figure 3(f) and Supplementary Fig. 2d. The effective stiffness *k* is the summation of the two elastic constants *k*_0_ and *k*_1_ shown in Figure 3(f) (also see Materials and Methods). The data shown in Figure 3(g) revealed higher stiffness in the distal region than in the middle region of the mandibular arch (i.e., heterogeneity).

To investigate stiffness anisotropy, the stiffness value was normalized by the minimum value of each embryo to exclude interembryo stiffness differences. In the middle region (Figure 3(h)), the stiffness values in all six directions were similar with no statistical difference (i.e., isotropy). In the distal region, the stiffness values in the proximal, distal, anterior, and posterior directions were similar. However, in the medial direction, a lower stiffness value was measured, and interestingly, a higher stiffness value was measured in the opposite, lateral direction, as shown in Figure 3(h). Figure 3(i) shows that the distal region of the mandibular arch has a higher anisotropic index, which is defined as the ratio of maximum stiffness and minimum stiffness, than the middle region which is close to isotropic (i.e., anisotropic index = 1). In summary, in the mouse mandibular arch, stiffness is spatially different (i.e., heterogeneous) from the middle region to the distal region and directionally dependent (i.e., anisotropic) in the distal region.

### 2.3. Fibronectin Expression Domain and Fiber Orientation Match Stiffness Heterogeneity and Anisotropy

**Figure 4:**
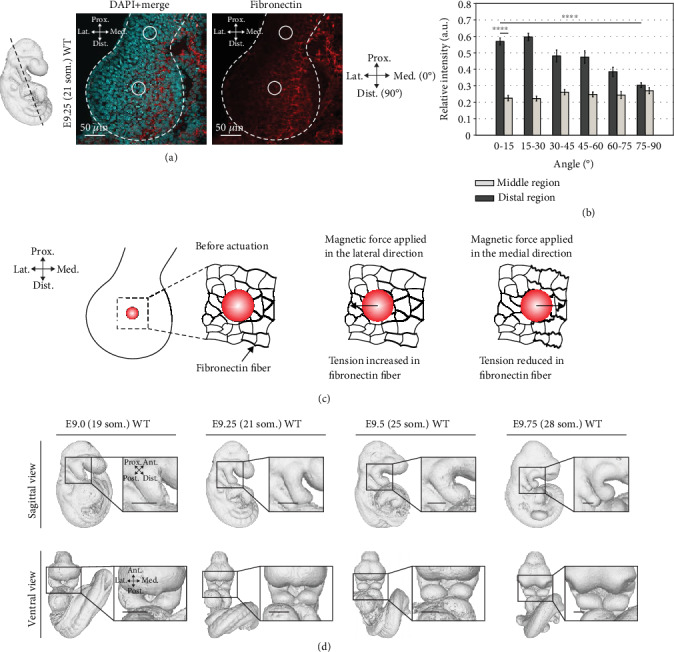
Fibronectin expression domain and fiber orientation match stiffness heterogeneity and anisotropy. (a) Transverse section of E9.25 (21 som.) WT embryos at mandibular region. Sections were stained with DAPI (cyan) and antifibronectin antibody (red). The black dashed line indicates the cryosection location. The white dashed lines outline the mandibular arch. The solid circles indicate the approximate location of the magnetic beads when performing stiffness measurement. (b) The angular distribution of fibronectin immunostaining fluorescence intensity (E9.25 (21 som.); *n* = 3 embryos) relative to the arch medial-lateral axis that was designated as 0 degree. Fluorescence intensity was quantified in the middle and distal regions using SIESTA (two-tailed *t*-test, ^∗∗∗∗^*P* < 0.0001). (c) Proposed mechanism of tension alteration by magnetic bead actuation, causing a lower stiffness value measured in the medial direction in the distal region. (d) WT mouse mandibular arch shape changes from E9.0 (19 som.) to E9.75 (28 som.) reconstructed from optical projection tomography. Error bars indicate s.e.m. Scale bars represent 200 *μ*m (d).

It has been shown that tissue stiffness is largely dominated by ECM compositions [16]. Our recent study revealed that a stiffness gradient in the mouse limb bud matches the fibronectin expression domain [7]. Since it was shown that fibronectin mRNA exhibited a distally biased localization in the mandibular arch [40], we examined the fibronectin expression by immunostaining. Immunofluorescence intensity of fibronectin was found to be spatially biased towards the distal region of the mandibular arch (Figure 4(a)); in agreement with tissue heterogeneity, we measured between the middle and distal regions. Unexpectedly, fibronectin was more abundant along the medial aspect of the arch, but by itself, that abundance does not explain anisotropic stiffness.

*In vitro*, it was shown that cells are stiffer along the predominant axis of stress fibers compared to the orthogonal direction [31, 32]. We therefore tested if the angular distribution of fibronectin is biased using a program called SIESTA [41]. Fluorescent fibronectin intensity was uniform in the middle region but biased along the lateral-medial axis in the distal region (Figure 4(b)). These findings correspond to the isotropic and anisotropic stiffness in those regions and suggest that the orientation of fibronectin cables underlies stiffness anisotropy.

## 3. Discussion

The ability to manipulate the direction of bead actuation along three orthogonal axes extends the repertoire of 3D multipole magnetic tweezers and fills an important gap among devices available to measure tissue properties. Constructing a device with a large workspace and long working distance in combination with deep tissue imaging was essential for examining anisotropic properties within the mandibular arch. By fairly simple application, multidirectional actuation allowed us to uncover unexpected properties which we use to generate hypotheses. The stiffness heterogeneity we identified (i.e., distal region is stiffer than the middle region) corresponds to distinct cell movements we reported previously. In particular, cell rearrangements are abundant within the relatively soft region and promote tissue streaming distally toward stiffer tissue where fibronectin is more abundant [14]. These observations suggest that the relative abundance of the extracellular matrix regulates the frequency of cell rearrangements and supports the possibility that durotaxis, or cell movement towards stiffer regions, orients collective cell movements *in vivo*.

The anisotropy index of 1.254 that we measured in the distal arch is within the range reported previously for cortical bone (ranging from ~1 to ~2.15) [42, 43] and corresponds to the distal mediolateral bias of fibronectin. Among cultured cells, cytoskeletal tension strongly influences the directional bias of stiffness [32], and the tension of fibrinous material may be a general mechanism that establishes stiffness anisotropy. In the arch, since distal beads were situated at the margin of the fibronectin domain, a potential explanation for the mediolateral anisotropy is that actuation away from the fibronectin domain stretches fibers, resulting in greater effective stiffness. When beads are actuated toward the fibronectin domain, they compress and reduce fiber pretension, resulting in lower stiffness (Figure 4(c)). A previous study has shown that fibronectin fibrils are easier to be compressed (folded) than stretched (unfolded) [44]. To build a causal relation between tension and stiffness, tension manipulation is required through mechanical, biochemical, and genetic approaches.

There are intriguing potential implications of stiffness anisotropy *in vivo*. Cell movements in the mandibular arch may include a mediolaterally biased component that complements and refines the collective movement of cells toward stiffer distal tissues. Axial tension or buckling of fibronectin cables may preferentially guide cell displacements, although it is not yet clear whether either mechanical process would promote or hinder cell movements. The medially biased abundance of fibronectin is also interesting since the left and right arches curve to join their distal projections at the midline (Figure 4(d)). Together with mediolaterally biased cables and stiffness anisotropy, that distribution may mechanically restrain medial elongation to promote curving of the lateral aspects of the two arches toward the midline.

Techniques for measuring tissue properties have advanced from 2D (i.e., epithelial sheets) to 3D (i.e., bulk mesenchyme) and from the surface of a tissue to deep within tissue. Our current magnetic tweezers enabled *in vivo* measurement of tissue properties at a depth and, uniquely, along six directions. Recent advances in deep and fast live imaging such as multiphoton scanned light-sheet microscopy [45–47] combined with our magnetic tweezers will permit deeper investigation of the dynamic role of tissue mechanical properties during development. Other potential applications include the precise application of forces to tissue in order to examine mechanisms of mechanotransduction *in vivo*, examination of tissue properties that correspond to disease states and development of phenotyping tests based on those properties. The device can potentially be scaled to accommodate more delicate embryonic samples or larger pathological specimens. By combining increasingly thorough measurement of mechanical properties and forces with live imaging and genetic and mechanical manipulation, one can open doors to previously unexplored mechanisms of morphogenesis and pathogenesis.

## 4. Materials and Methods

### 4.1. Mouse Strains

Analysis was performed using the following mouse strains: mTmG [48] (Jackson Laboratory: Gt(ROSA)26Sortm4(ACTB-tdTomato-EGFP)Luo/J)) and pCX-NLS-Cre [39] (Jackson Laboratory: NMRI.Cg-Tg(CAG-cre)1Nagy/Cnbc). All strains were outbred to CD1. All animal experiments were performed in accordance with protocols approved by the Animal Care Committee of the Hospital for Sick Children Research Institute.

### 4.2. Optical Projection Tomography

Mouse embryos were harvested and fixed in 4% paraformaldehyde overnight at 4°C. OPT was performed using a system that was custom-built and is fully described elsewhere [49]. Three-dimensional datasets were reconstructed from autofluorescence projection images acquired during a 25 min. Scan period was at an isotropic voxel size of 4.5 *μ*m. The mandibular arch structure was segmented from the embryo and rendered in MeshLab.

### 4.3. Magnetic Tweezer Device

Uniform magnetic field gradient was generated by an eight-pole magnetic device. The magnetic poles and pole holders were machined by computer numerical control (CNC) from low carbon steel 1018 and aluminum, respectively. Each core was wound 500 times with American wire gauge 24 copper wire. A customized sample rotation stage was 3D printed from polylactic acid (PLA). The device stage and sample stage were laser cut from 5.6 mm and 1.6 mm thick acrylic sheets, respectively. The magnetic device was powered using a lab bench DC power supply. The four coils were series connected with four standard DPDT switches to control current direction. The magnetic device was mounted on a Leica two-photon confocal system (Leica DM6000) that supports a live imaging chamber. The maximum penetration depth of two-photon microscopy as well as the maximum embedded depth of the magnetic beads was no less than 1 mm.

### 4.4. Magnetic Field Simulation

Magnetic field simulation of the device was performed in COMSOL Multiphysics 5.4 (COMSOL Inc.). The HB (magnetization) curves of silicon iron and LC steel 1018 were imported into the software and assigned to the poles. A sweep function (from 1 A to 5 A with 0.2 A step size) was incorporated in the simulation to derive the magnetic field under different driving currents to determine the minimum current to saturate the magnetic beads. At 3 A driving current, the lowest magnetic field in the working space was determined to be 50.3 mT as shown in Figure 2(c), which is sufficient to saturate the magnetic bead according the bead magnetization curve (Spherotech, SVMH-400-4).

### 4.5. Magnetic Force Calibration

To calibrate the magnetic force generated by our magnetic device, magnetic beads (Spherotech, SVMH-400-4) were dispersed in silicone oil (Sigma-Aldrich) of known viscosity. In detail, 5 *μ*L of beads and 1 mL of silicone oil were placed in an Eppendorf tube. To avoid bead aggregation, ultrasound (Model 60, Fisher Scientific) was used to fully mix the solution. The bead-silicone oil solution was placed in a 30 mm petri dish. After a few minutes when all flows in the solution were settled, the beads were actuated with bead movements recorded. Bead velocities were calculated using a customized MATLAB R2019b (MathWorks) program. Force exerted on the beads was calculated using the Stokes equation.

### 4.6. Magnetic Bead Functionalization and Microinjection

The streptavidin-coated Spherotech SVMH-400-4 superparamagnetic beads (diameter: 32 *μ*m) were coupled with Atto 565-biotin (Sigma-Aldrich) and biotinylated poly-L-lysine molecules through the streptavidin-biotin reaction. We placed 5 *μ*L streptavidin-coated magnetic bead solution into an Eppendorf tube. The bead solution was washed three times with phosphate-buffered saline (PBS) to remove preservatives. A permanent magnet was placed under the tube to collect the magnetic beads. The beads were then collected by a micropipette and resuspended in 90 *μ*L of Milli-Q. Atto 565-biotin (1 mg) was diluted in 200 *μ*L of ethanol. Five *μ*L of this dilution and 5 *μ*L of the biotinylated adhesion molecule solution (1 : 1000 in PBS) were mixed with 90 *μ*L of the magnetic bead suspension for 30 minutes. Finally, the solution was washed with PBS five times to remove the biotin surplus through magnetic separation, and the supernatant was collected using a micropipette.

In microinjection, a microneedle pulled from a glass capillary tube using a micropipette laser puller was loaded with mineral oil. One magnetic bead each time was aspirated into the microneedle using a microinjector (CellTram 4r Oil, Eppendorf) and then injected into the center of the middle and distal regions of the mouse mandibular arch.

### 4.7. Cell Apoptosis Detection

LysoTracker Red DND-99 (Thermo Fisher) was diluted to 2 *μ*M in DMEM containing 50% rat serum. Embryos were placed in the medium and incubated in a roller culture apparatus for 1 h. The temperature was maintained at 37°C with 5% CO_2_. Embryos were washed three times with PBS after staining to remove the LysoTracker surplus, then fixed overnight in 4% paraformaldehyde in PBS followed by 3 washes in PBS. Images were acquired using a Nikon A1R Si Point Scanning Confocal microscope at 20x magnification, and analysis was performed using ImageJ.

### 4.8. Polyacrylamide Gel Verification Experiment

Polyacrylamide (PA) gels were prepared by mixing acrylamide (3%, Bio-Rad) and bisacrylamide (0.01% Bio-Rad) in deionized water. Polymerization was initiated with 0.05% ammonium persulfate (Sigma) and 0.1% N,N,N′,N′-tetramethylethylenediamine (TEMED, Sigma). Five *μ*L of magnetic (Spherotech, SVMH-400-4) and nonmagnetic fluorescent (F8842, Thermo Fisher) beads in deionized water (1 : 100) were added to 150 *μ*L PA gel solution. The PA gels containing magnetic fluorescent beads and PA gels with nonmagnetic fluorescent beads were actuated in the magnetic tweezer device, and bead displacements were recorded by two-photon confocal microscopy at 2 Hz. The bead displacement (in the direction of the magnetic force) was tracked with subpixel resolution.

### 4.9. Stiffness Quantification *In Vivo*

Embryos with magnetic beads injected were suspended in a solution of DMEM without phenol red containing 12.5% rat serum and 1% low-melt agarose (Invitrogen) in a glass capillary tube. Once the agarose solidified, the agarose plug was partially extruded from the glass capillary tube onto the sample stage until the portion containing the embryo was completely outside of the capillary. Magnetic beads were actuated by the magnetic device in the proximal, distal, anterior, and posterior directions in the sagittal view. The sample was then rotated 90 degrees to the dorsal side, and lateral and medial actuations were performed. The bead displacements were recorded by the two-photon confocal microscopy at 2 Hz. The bead displacement (in the direction of the magnetic force) was tracked with subpixel resolution and fitted using a viscoelastic model in MATLAB to extract stiffness values.

As shown in Figure 3(f), the displacement-time data reveals an immediate elastic response followed by a continuous creep and the displacement plateaus near the end of force application. The standard linear solid model, which is commonly applied to interpret tissue viscoelastic properties [6], was used to extract viscoelastic parameters from the experimental data, as illustrated in Supplementary Fig. 2d. Briefly, The displacement/force-time relationship of the standard linear solid model is

(1) $$\begin{array}{c} \frac{x\left(t\right)}{F}=\frac{1}{k_{0}}\left(1-\frac{k_{1}}{k_{0}+k_{1}}e^{-\left(t/\tau \right)}\right);\tau =\frac{\mu \left(k_{0}+k_{1}\right)}{k_{0}k_{1}}, \end{array}$$

where *x*(*t*) is the displacement of the bead at time *t*, *F* is the applied force, *k*_0_ and *k*_1_ are the two elastic constants, *μ* is the effective viscosity, and *τ* is the relaxation time. The effective stiffness *k* (unit: Pa·m), *k* = *k*_0_ + *k*_1_, can be calculated from the immediate elastic response (segment (1) in Supplementary Fig. 2d),

(2) $$\begin{array}{c} k=\frac{1}{\text{height of the elastic response}}. \end{array}$$

The elastic constants *k*_0_ can be determined from the plateau height as

(3) $$\begin{array}{c} k_{0}=\frac{1}{\text{height of the plateau}}. \end{array}$$

The effective viscosity *μ* (unit: Pa·s·m) can be extracted by fitting the continuous creep (segment (2) in Supplementary Fig. 2d).

### 4.10. Subpixel Tracking

To capture bead displacement with a subpixel resolution, the edge of the bead was determined by applying a subpixel edge detector [50] on the grayscale image. The detector [50] was used in this work because of its high accuracy and robustness to image noise compared to other detectors such as moment-based [51], least-square-error-based [52], and interpolation-based detectors [53]. Briefly, the intensity *F*_*i*,*j*_ of a pixel (*x*, *y*) on the edge is

(4) $$\begin{array}{c} F_{i,j}=\frac{AS_{A}+BS_{B}}{S_{A}+S_{B}}, \end{array}$$

where *A* and *B* are the intensities at the two sides of the edge and *S*_*A*_ and *S*_*B*_ are the areas of that pixel covered by intensities *A* and *B*, respectively. The edge is approximated by a second-degree polynomial function *y* = *a* + *bx* + *cx*^2^. The subpixel position of the edge is obtained from the coefficients *a*, *b*, and *c*, which are solved by considering the intensities of neighbor pixels. The algorithm achieved an accuracy of 0.2 pixels (pixel size: 0.37 *μ*m) as evaluated using synthetic test images with artificially generated circles and as validated by tracking magnetic beads embedded in polyacrylamide (PA) gel (Supplementary Fig. 2b). This technique was used to track the displacement of the magnetic beads.

### 4.11. Immunofluorescence

Dissected mouse embryos were fixed overnight in 4% paraformaldehyde in PBS followed by 3 washes in PBS. Fixed embryos were embedded in 7.5% gelatin/15% sucrose and sectioned into 10 *μ*m slices using a Leica CM1800 cryostat. Sections were washed 2 × 5 min in Milli-Q and 1 × 5 min in PBS, permeabilised in 0.1% Triton X-100 in PBS for 20 min, and blocked in 5% normal donkey serum (in 0.05% Triton X-100 in PBS) for 1 h. Sections were incubated in a primary antibody overnight at 4°C followed by 4 × 10 min washes in 0.05% Triton X-100 in PBS, then incubated in a secondary antibody (1 : 1000) for 1 h at room temperature. Finally, sections were washed 3 × 5 min in 0.05% Triton X-100 in PBS and 2 × 5 min in PBS. Images were acquired using a Nikon A1R Si Point Scanning Confocal microscope, and fluorescent analysis was performed using SIESTA [41].

### 4.12. Antibodies

The antibody used was anti-fibronectin (Abcam, 1 : 100). All secondary antibodies were purchased from Jackson Immunoresearch and used at 1 : 1000 dilutions.

## Supplementary Materials

Supplementary Materials Supplementary Figure 1: temperature change in the workspace during magnetic actuation. (a) Temperature change in the workspace center under 10 s (inset) of 5 A actuation. Error bars indicate s.d. Supplementary Figure 2: mandibular arch stiffness quantification. (a) Confocal images of E9.25 WT mandibular arches visualized with LysoTracker (red) with and without magnetic beads injected. (b) Subpixel tracking of the position of a magnetic bead embedded in polyacrylamide (PA) gel. (c) Displacement of the magnetic and fluorescent beads embedded in PA gel during one actuation cycle (inset). (d) Theoretical creep response and relaxation curve of the standard linear solid model.

## References

[1] Thompson D. W., Bonner J. T. (2014). *On growth and form*. *On Growth and Form*.

[2] Heisenberg C. P., Bellaïche Y. (2013). Forces in tissue morphogenesis and patterning. *Cell*.

[3] Bi D., Lopez J. H., Schwarz J. M., Manning M. L. (2015). A density-independent rigidity transition in biological tissues. *Nature Physics*.

[4] Hutson M. S., Tokutake Y., Chang M. S. (2003). Forces for morphogenesis investigated with laser microsurgery and quantitative modeling. *Science*.

[5] Lau K., Tao H., Liu H. (2015). Anisotropic stress orients remodelling of mammalian limb bud ectoderm. *Nature Cell Biology*.

[6] Serwane F., Mongera A., Rowghanian P. (2017). In vivo quantification of spatially varying mechanical properties in developing tissues. *Nature Methods*.

[7] Zhu M., Tao H., Samani M. (2020). Spatial mapping of tissue properties in vivo reveals a 3D stiffness gradient in the mouse limb bud. *Proceedings of the National Academy of Sciences of the United States of America*.

[8] Maitre J.-L., Berthoumieux H., Krens S. F. G. (2012). Adhesion functions in cell sorting by mechanically coupling the cortices of adhering cells. *Science*.

[9] Mongera A., Rowghanian P., Gustafson H. J. (2018). A fluid-to-solid jamming transition underlies vertebrate body axis elongation. *Nature*.

[10] Skoglund P., Rolo A., Chen X., Gumbiner B. M., Keller R. (2008). Convergence and extension at gastrulation require a myosin IIB-dependent cortical actin network. *Development*.

[11] Green J. B. A., Davidson L. A. (2007). Convergent extension and the hexahedral cell. *Nature Cell Biology*.

[12] Munjal A., Philippe J. M., Munro E., Lecuit T. (2015). A self-organized biomechanical network drives shape changes during tissue morphogenesis. *Nature*.

[13] Shindo A., Inoue Y., Kinoshita M., Wallingford J. B. (2019). PCP-dependent transcellular regulation of actomyosin oscillation facilitates convergent extension of vertebrate tissue. *Developmental Biology*.

[14] Tao H., Zhu M., Lau K. (2019). Oscillatory cortical forces promote three dimensional cell intercalations that shape the murine mandibular arch. *Nature Communications*.

[15] Tada M., Heisenberg C. P. (2012). Convergent extension: using collective cell migration and cell intercalation to shape embryos. *Development*.

[16] Swift J., Ivanovska I. L., Buxboim A. (2013). Nuclear lamin-A scales with tissue stiffness and enhances matrix-directed differentiation. *Science*.

[17] Wen J., Tao H., Lau K. (2017). Cell and tissue scale forces coregulate Fgfr2-dependent tetrads and rosettes in the mouse embryo. *Biophysical Journal*.

[18] Koser D. E., Thompson A. J., Foster S. K. (2016). Mechanosensing is critical for axon growth in the developing brain. *Nature Neuroscience*.

[19] Barriga E. H., Franze K., Charras G., Mayor R. (2018). Tissue stiffening coordinates morphogenesis by triggering collective cell migration in vivo. *Nature*.

[20] Saez A., Ghibaudo M., Buguin A., Silberzan P., Ladoux B. (2007). Rigidity-driven growth and migration of epithelial cells on microstructured anisotropic substrates. *Proceedings of the National Academy of Sciences of the United States of America*.

[21] Islam A., Younesi M., Mbimba T., Akkus O. (2016). Collagen substrate stiffness anisotropy affects cellular elongation, nuclear shape, and stem cell fate toward anisotropic tissue lineage. *Advanced Healthcare Materials*.

[22] Zhang H., Lin F., Huang J., Xiong C. (2020). Anisotropic stiffness gradient-regulated mechanical guidance drives directional migration of cancer cells. *Acta Biomaterialia*.

[23] Efremov Y. M., Velay-Lizancos M., Weaver C. J. (2019). Anisotropy vs isotropy in living cell indentation with AFM. *Scientific Reports*.

[24] Goto T., Davidson L., Asashima M., Keller R. (2005). Planar cell polarity genes regulate polarized extracellular matrix deposition during frog gastrulation. *Current Biology*.

[25] Neuman K. C., Nagy A. (2008). Single-molecule force spectroscopy: optical tweezers, magnetic tweezers and atomic force microscopy. *Nature Methods*.

[26] Dai J., Sheetz M. P. (1995). Mechanical properties of neuronal growth cone membranes studied by tether formation with laser optical tweezers. *Biophysical Journal*.

[27] Nawaz S., Sánchez P., Bodensiek K., Li S., Simons M., Schaap I. A. T. (2012). Cell visco-elasticity measured with AFM and optical trapping at sub-micrometer deformations. *PLoS One*.

[28] Mandal K., Asnacios A., Goud B., Manneville J.-B. (2016). Mapping intracellular mechanics on micropatterned substrates. *Proceedings of the National Academy of Sciences*.

[29] Bambardekar K., Clément R., Blanc O., Chardès C., Lenne P.-F. (2015). Direct laser manipulation reveals the mechanics of cell contacts in vivo. *Proceedings of the National Academy of Sciences*.

[30] Chowdhury F., Na S., Li D. (2010). Material properties of the cell dictate stress-induced spreading and differentiation in embryonic stem cells. *Nature Materials*.

[31] Bonakdar N., Gerum R., Kuhn M. (2016). Mechanical plasticity of cells. *Nature Materials*.

[32] Hu S., Eberhard L., Chen J. (2004). Mechanical anisotropy of adherent cells probed by a three-dimensional magnetic twisting device. *American Journal of Physiology-Cell Physiology*.

[33] Bausch A. R., Möller W., Sackmann E. (1999). Measurement of local viscoelasticity and forces in living cells by magnetic tweezers. *Biophysical Journal*.

[34] Wang X., Ho C., Tsatskis Y. (2019). Intracellular manipulation and measurement with multipole magnetic tweezers. *Science robotics*.

[35] D’Angelo A., Dierkes K., Carolis C., Salbreux G., Solon J. (2019). In vivo force application reveals a fast tissue softening and external friction increase during early embryogenesis. *Current Biology*.

[36] Wang X., Luo M., Wu H. (2018). A three-dimensional magnetic tweezer system for intraembryonic navigation and measurement. *IEEE Transactions on Robotics*.

[37] Wang X., Zhang Z., Tao H., Liu J., Hopyan S., Sun Y. (2018). Characterizing inner pressure and stiffness of trophoblast and inner cell mass of blastocysts. *Biophysical Journal*.

[38] Yarmolenko P. S., Moon E. J., Landon C. (2011). Thresholds for thermal damage to normal tissues: an update. *International Journal of Hyperthermia*.

[39] Belteki G. (2005). Conditional and inducible transgene expression in mice through the combinatorial use of Cre-mediated recombination and tetracycline induction. *Nucleic Acids Research*.

[40] Mittal A., Pulina M., Hou S. Y., Astrof S. (2010). Fibronectin and integrin alpha 5 play essential roles in the development of the cardiac neural crest. *Mechanisms of Development*.

[41] Fernandez-Gonzalez R., Zallen J. A. (2011). Oscillatory behaviors and hierarchical assembly of contractile structures in intercalating cells. *Physical Biology*.

[42] Espinoza Orías A. A., Deuerling J. M., Landrigan M. D., Renaud J. E., Roeder R. K. (2009). Anatomic variation in the elastic anisotropy of cortical bone tissue in the human femur. *Journal of the Mechanical Behavior of Biomedical Materials*.

[43] Abdel-Wahab A. A., Alam K., Silberschmidt V. V. (2011). Analysis of anisotropic viscoelastoplastic properties of cortical bone tissues. *Journal of the Mechanical Behavior of Biomedical Materials*.

[44] Erickson H. P. (2002). Stretching fibronectin. *Journal of Muscle Research & Cell Motility*.

[45] Truong T. V., Supatto W., Koos D. S., Choi J. M., Fraser S. E. (2011). Deep and fast live imaging with two-photon scanned light-sheet microscopy. *Nature Methods*.

[46] Tomer R., Khairy K., Amat F., Keller P. J. (2012). Quantitative high-speed imaging of entire developing embryos with simultaneous multiview light-sheet microscopy. *Nature Methods*.

[47] McDole K., Guignard L., Amat F. (2018). In toto imaging and reconstruction of post-implantation mouse development at the single-cell level. *Cell*.

[48] Muzumdar M. D., Luo L., Zong H. (2007). Modeling sporadic loss of heterozygosity in mice by using mosaic analysis with double markers (MADM). *Proceedings of the National Academy of Sciences*.

[49] Wong M. D., Dazai J., Walls J. R., Gale N. W., Henkelman R. M. (2013). Design and implementation of a custom built optical projection tomography system. *PLoS One*.

[50] Trujillo-Pino A., Krissian K., Alemán-Flores M., Santana-Cedrés D. (2013). Accurate subpixel edge location based on partial area effect. *Image and Vision Computing*.

[51] Da F., Zhang H. (2010). Sub-pixel edge detection based on an improved moment. *Image and Vision Computing*.

[52] Ye J., Fu G., Poudel U. P. (2005). High-accuracy edge detection with blurred edge model. *Image and Vision Computing*.

[53] Hermosilla T., Bermejo E., Balaguer A., Ruiz L. A. (2008). Non-linear fourth-order image interpolation for subpixel edge detection and localization. *Image and Vision Computing*.

