# Supplementary figures and images for: Magnetic Micromanipulation for In Vivo Measurement of Stiffness Heterogeneity and Anisotropy in the Mouse Mandibular Arch
Source: Research (Wash D C). 2020 Jun 22;2020:7914074. doi: 10.34133/2020/7914074 (PMC7327709; doi:10.34133/2020/7914074)

Supplementary Figure 1

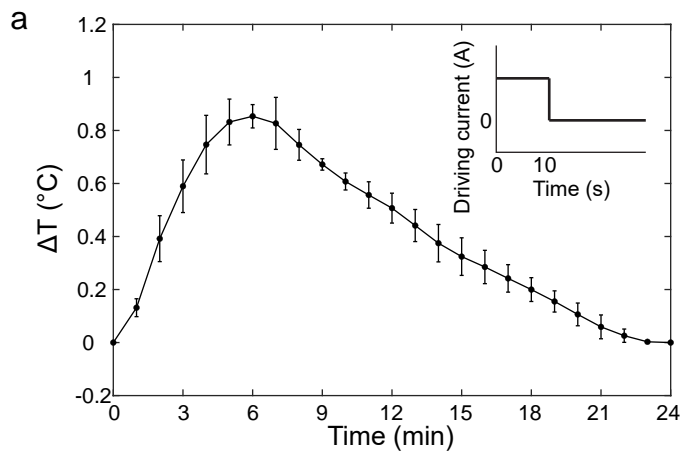

Supplementary Figure 2

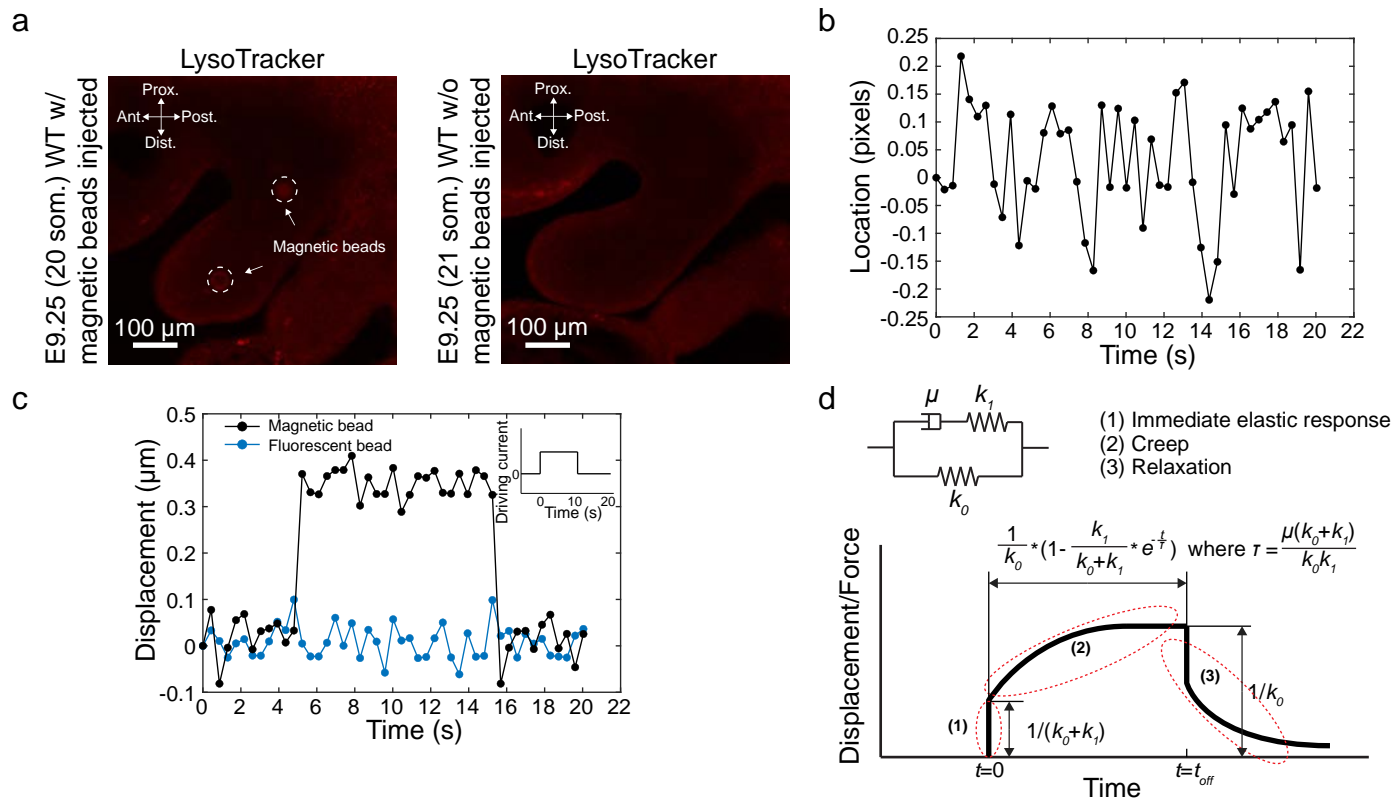

Supplement: Supplementary Materials — Supplementary Figure 1: temperature change in the workspace during magnetic actuation. (a) Temperature change in the workspace center under 10 s (inset) of 5 A actuation. Error bars indicate s.d. Supplementary Figure 2: mandibular arch stiffness quantification. (a) Confocal images of E9.25 WT mandibular arches visualized with LysoTracker (red) with and without magnetic beads injected. (b) Subpixel tracking of the position of a magnetic bead embedded in polyacrylamide (PA) gel. (c) Displacement of the magnetic and fluorescent beads embedded in PA gel during one actuation cycle (inset). (d) Theoretical creep response and relaxation curve of the standard linear solid model. [file 7914074.f1.pdf]
